# Form and Function in Information for Visual Perception

**DOI:** 10.1177/20416695211053352

**Published:** 2021-12-23

**Authors:** Joseph S. Lappin, Herbert H. Bell

**Affiliations:** 5718 Vanderbilt University, Nashville TN, United States

**Keywords:** 3D perception, attention, spatial vision, capacity/resolution, stereopsis, motion, optic flow, temporal processing, shape, spatiotemporal factors

## Abstract

Visual perception involves spatially and temporally coordinated variations in diverse physical systems: environmental surfaces and symbols, optical images, electro-chemical activity in neural networks, muscles, and bodily movements—each with a distinctly different material structure and energy. The fundamental problem in the theory of perception is to characterize the information that enables both perceptual awareness and real-time dynamic coordination of these diverse physical systems. Gibson's psychophysical and ecological conception of this problem differed from that of mainstream science both then and now. The present article aims to incorporate Gibson's ideas within a general conception of information for visual perception. We emphasize the essential role of spatiotemporal form, in contrast with symbolic information. We consider contemporary understanding of surface structure, optical images, and optic flow. Finally, we consider recent evidence about capacity limitations on the rate of visual perception and implications for the ecology of vision.

The basic concern of psychophysics is the nature of information for perception. Gibson’s (1950) theory of visual perception was based on psychophysics:

*The first place to look is obviously the retinal image. If, contrary to past teaching, there are exact concomitant variations in the image for the important features of the visual world a psychophysical theory will be possible.* (p. 61)

Stevens (1951) offered a related assessment:

*In a sense there is only one problem of psychophysics, namely, the definition of the stimulus. In this same sense there is only one problem in all of psychology — and it is the same problem.* (p.31)

When Gibson and Stevens made these statements, scientific psychology was still often considered a study of ‘responses’ to ‘stimuli’. A stimulus was the cause, and a response was the effect. Neither term, however, was objectively defined or consistently used. Gibson (1960) pointed out that the word ‘stimulus’ had so many different meanings that its explanatory value was obscure. He concluded his review of ambiguities of the ‘stimulus’ concept with a proposal that “We need to know the laws of stimulus information” (p. 702). ‘Stimulus’ and ‘information’ entail different properties, however.

Neither Gibson nor Stevens had a good alternative to the term ‘stimulus’ for the input to perception and cognition. And related problems with terminology persist! How indeed can we understand causal relations between such distinct physical domains as the distal environment, proximal sensory stimulation, electro-chemical changes in dense neural networks, conscious experience, concepts, and actions? How do changing environmental conditions affect behavioral actions in real time? The traditional intuitive concepts of cause and effect seem insufficient.

Whereas the term ‘stimulus’ suggests passive processes of sensation, perception, and response, ‘information’ suggests selective observation and purposive actions. Both terms, however, have vague usage. Both have been generic labels for the apparent antecedents of widely varied behaviors. ‘Information’ has referred to the input for perceptual processes from detection to visual-motor coordination and for cognitive processes from memory to problem solving. ‘Information’ might refer to a discrete object or to a spatiotemporal pattern of energy, in the distal environment or at the proximal senses. Because the concept of information is essential to theories of perception, and because the term has varied meanings, a review of basic principles is useful.

Two general issues include: (1) What is information—as a general concept, and for particular instances of perception and action? (2) How much information can be acquired, as a general limiting parameter of perception and performance? Gibson's perceptual research focused on the psychophysics of perceptual information, with sparse attention to the limitations of perception. Mainstream psychology, however, has often focused on the limitations of perception, though often without a clear or consistent definition of information. The following sections consider basic aspects of the concept of information.

## Information Involves Dyadic Relations. It is not an Objective Thing

If the input to perception is information, then it is natural to think of it as something objective and quantifiable, independent of an observer. In technology, information is often treated as specific signals sent from a source to a destination. And observers might be said to perceive environmental information. Natural as such ideas may seem, they are misleading.

A basic but counterintuitive property of information is that it involves a dyadic *relationship* between two separate subsystems—a correlation of corresponding variations in physically separate systems. Shannon’s (1948) mathematical theory of communication is often treated as the principal foundation for “information theory”, but it was not a theory of information as such. Rather, as Shannon emphasized, it was a theory of *communication*, of a correlation between a sender and a receiver. In that general sense, information involves a dyadic relation, a correlation, with corresponding variations in two or more separate domains. Treating information as an objective property or thing in any single domain causes confusions.

Gibson’s (1979) ecological approach emphasized a similar idea about relationships inherent in the concepts of environment, perception, and action: “. . . the words *animal* and *environment* make an inseparable pair. Each term implies the other” (p. 8). “The environment of animals and men is what they perceive. The environment is not the same as the physical world, . . .” (p. 15). Eleanor Gibson developed a similar idea in her book on *Perceptual learning and development* (1969): “. . . the information in the structure of stimulation is potential; it is not necessarily picked up” (p. 14).

Thus, we may identify environmental patterns that can guide an observer's perceptions and actions, but these are *potential* information. Various patterns may or may not be used or even noticed by a particular observer in guiding actions for particular purposes. The same scenario offers different information for different observers, depending on the perspectives afforded by their locations, purposes, interests, and knowledge of context.

Accordingly, Gibson consistently emphasized that the perceived environment of any given observer is inherently meaningful. Chapter three of *The Ecological Approach* (1979), on “The meaningful environment,” begins with the following point:

*If what we perceived were the entities of physics and mathematics, meanings would have to be imposed on them. But if what we perceive are the entities of environmental science, their meanings can be* discovered *(p. 33)*.

In contrast, meaning is supposedly irrelevant in Shannon's mathematical theory. His theory gives a general method for quantifying a correlation between a source and destination, abstracted from the specific forms of variation and from the particular functions and meanings of information. An implicit property of Shannon's system, however, is that the possible signals are known beforehand by both sender and receiver. Thus, meaning is inherent in Shannon's system before information is transmitted.

In Shannon's system, information can be quantified by reduced uncertainty. A general concept of information, however, should not rest on anticipation and quantifiable uncertainty. In contrast, in Gibson's psychophysical and ecological approach, spatiotemporal images convey information by structural correspondences with environmental objects and events. For Gibson, the structure is the meaning.

Another form of information is encountered in the information-processing paradigm in psychology, usually consisting of discrete objects, features, or alphanumeric symbols. Experimental psychology in the second half of the 20th century was freed from the limited causal relations implicit in concepts of ‘stimuli’ and ‘responses’. Human perception and cognition were often re-envisioned by analogy with machines for processing information. Information in machines is usually represented by digital symbols, and symbolic representation is usually implicit in the information-processing paradigm. Spatiotemporal structure usually is irrelevant in that paradigm.^1^

Information is not necessarily symbolic, however. Fundamentally, information is based on corresponding variations in two physically separate domains. Psychophysical information in Gibson's ecological psychology is primarily spatiotemporal.

## A Psychophysical Continuum From Spatiotemporal to Symbolic Information

Structural correspondences between proximal images and their distal environmental referents vary on a continuum from natural spatiotemporal similarity at one end to abstract human-made artifacts at the other, from optical images of environmental surfaces in the eyes of active observers to alphanumeric characters and other symbols. The contrast between the two extremes is the principal present concern, but a rich variety of artifacts lies between. Consider the many varieties of abstract paintings (impressionism, cubism, etc.), silhouettes, sketches, cartoons, and pictograms. Even realistic portraits, photographs, and movies extract distinguishing features from typical appearances and context. Frames, galleries, and theaters make such abstraction explicit. Information in general, from spatiotemporal to symbolic, is selective. So is perception.

### The representational theory of measurement

The logic of how variations in one domain constitute information about those in another domain is exemplified by the representational theory of measurement (e.g., Krantz, Luce, Suppes, & Tversky, 1971; Roberts, 1979; Suppes & Zinnes, 1963). A brief discussion is relevant because the logic of numerical representation resembles that of information in general. Measurement is said to consist of two fundamental problems: the *representation problem* and the *uniqueness problem*, roughly analogous to semantics and syntax.

The representation problem is to identify the qualitative relational structure of a set of empirical observations and show that this structure is isomorphic to a particular structure of numerical relations. Importantly, the isomorphism is defined on the *relations* rather than the objects. For example, using a balance pan to evaluate weights involves qualitative binary relations of inequality and equality and a concatenation operation for combining multiple objects in the same pan. Those qualitative relations may be represented by numerical relations of ***<*** and ***=*** and a summation operation, ***+***. Thus, if aεb is the qualitative relation ‘ *a* equals b ’, and if ‘*a* ⋏ *b*’ is the concatenation operation, then numerical representation in the real numbers, ℜ, is an isomorphism of ternary relations such as ‘*a* ⋏ *b* εc ’ if and only if *x*  *+*  *y*  *=*  *z*, for all a,b , c *∈ A* and all real numbers *x, y, z* ∈ ℜ.

Information often involves correspondences that are not strictly isomorphic, but the logic is similar: Information requires corresponding relational structures. The correspondence is between the relationships, not individual objects or symbols. This principle applies in particular to visual perception, where spatiotemporal structures correspond across environment, optical images, neural activity patterns, etc.

Measurement theory is said to involve a second fundamental problem, concerning uniqueness of the numerical assignment. This is an invariance issue: What numerical transformations preserve isomorphism with the empirical relations? Thus, a ratio scale is invariant under multiplication by a scalar constant. Ratios of distances are equivalent whether measured in meters, feet, or inches. Relations on interval scales are invariant under linear transformations of the numerical scale, and nominal-scale representations are invariant under any transformation that preserves the empirical categories.

*Invariance of corresponding relational structure is the foundation of all information*. The relevant relational structure in any domain is *defined by* the transformations under which that relational structure is invariant. In comparing objects on a balance scale, for example, the relevant property is identified by the physical transformations that preserve those comparative relations. Relative weight is obviously invariant with changes in shape, volume, material substance, color, etc.; and the concept of mass is identified by invariance of the balance relations under changes in the gravitational field or acceleration.

Similarly, visual information about environmental structure requires invariance of optical image relations involving surface shapes, colors, motions, etc. under changes in both the observer's relative position and motion within the environment and changes in environmental conditions such as ambient illumination and surrounding context.

### Symbolic versus spatiotemporal information

By definition, the informational significance of a symbol is unrelated to its spatial and temporal form. The physical forms serve merely to distinguish between symbols. In the information-processing paradigm of 20th-century psychology, sensory information often resembled what it had been before: Sensory ‘stimuli’ and even neural ‘spikes’ could be construed as categorical symbols to be transformed or processed. Hypothesized systems for processing symbolic information were much more complex than mere ‘responses’, but the input for perception and cognition was often a categorical event. Spatiotemporal structure was usually irrelevant.

In contrast, spatiotemporal form was essential to Gibson's ecological approach. From his 1950 book onward, Gibson sought to elucidate corresponding structures of environmental and sensory patterns. He pointed out that observers’ perceptions and actions coordinated in real time with the dynamic structures of their environments because spatiotemporal variations of sensory patterns corresponded to those of the environment. Texture density gradients, for example, were considered information about surface slant.^2^ Corresponding spatiotemporal structures in distal environments and proximal stimulation evidently were both available and necessary to support real-time coordination of actions with environments. Thus, the senses could be considered as perceptual systems. And one could regard environments as perceived “directly”, rather than inferred logically by combining various sensory cues and prior knowledge.

### Symbols and spatiotemporal forms require different perceptual systems

In Wiener’s (1948/1961) *Cybernetics*, information was spatiotemporal, suitable for guiding the actions of animals and analog machines. Computer engineering, however, has been dominated by digital technology. Shannon's quantification of information by binary “bits”, *0* versus *1*, was useful in engineering digital systems.

The idea that human perception and cognition can be considered symbol-processing systems has appealed to many scientists. Newell and Simon’s (1972) volume on *Human problem solving* explicates the rationale for studying human problem solving as symbol processing. A similar rationale is implicit in other research on perception and cognition.

From the standard information-processing perspective, visible patterns are often represented as configurations of discrete elements — cues, features, objects, edges, letters, etc. Physical definitions of such sensory elements are often arbitrary, based on properties convenient for experimental manipulation. Spatiotemporal relations among such symbolic elements usually are not explicit.

If the forms of symbols and their referents are unrelated, then the functional significance of any particular symbol pattern necessarily originates in the observer's processes for relating current and past input patterns. Such processes are then needed to detect, encode, group, integrate, categorize, interpret, and store that information. Observers’ schemas and Bayesian statistical inference are needed to interpret the environmental correlates of the sensory data patterns. Semantics and syntax of symbol systems often depend on the community of users.

In theories of human and machine vision, spatial positions and relations are sometimes described by reference to extrinsic coordinates defined by retinal anatomy or photosensor array hardware. Marr (1982), for example, said it was obvious that the spatial structure of retinal images constitutes a scalar field with retinal coordinates. This representation of optical information separates the spatiotemporal structure of images from that of the environment, making the visual system responsible for recovering the environmental geometry. As a result, visual perception is said to be an “ill-posed” problem—requiring inferences based on prior and statistical knowledge about the environmental sources of limited image information. The ill-posed problem, however, is that framed by this representation of image information.

The insufficiency of representing image information as two-dimensional (2-D) scalar fields may be seen by considering the image transformations associated with active observers in dynamic environments. Optical images are continually transformed by (a) observers’ viewing positions, (b) environmental object motions, (c) ambient illumination, (d) environmental context, and (e) observers’ prior knowledge. Image information about the environmental correlates must, therefore, be invariant or at least stable under those transformations.

If corresponding spatiotemporal structures of optical images and environments can be identified, then Gibson’s (1966) consideration of the senses as perceptual systems and his (1979) ecological approach are plausible, parsimonious, and fruitful. This psychophysical approach does not mean that observers’ neural or computational processes for receiving sensory information are irrelevant. Instead, these processes *reveal* rather than supply the coherence of sensory information. Nor does the psychophysical approach mean that learning and past experience are irrelevant. Experienced observers learn how things vary (Gibson & Gibson, 1955). Expertise enables recognition of relevance and meaning (e.g., Chase & Simon, 1973).

Neural and computational systems need to preserve real-time spatiotemporal coherence of sensory input. Descriptions of neural networks and brain functions, however, often involve encoding and integration of input features and supplementary association with previous patterns. Temporal processes of cortical physiology are also described as accumulating different information at different rates in different cortical areas. How exactly such neural systems might preserve coherent forms of sensory information about dynamic environments is not yet clear.

A mathematical model of cortical function recently developed by Wason (2021) suggests how brain activity might preserve the spatiotemporal coherence. Specifically, cortical physiology and columnar architecture can support dynamic information transmission by coherent phase relations among distributed neural processes. Neural networks in this model function as coherent apertures, similar to phased-array radar systems and holography. A coherent aperture maintains high mutual information between inputs and outputs and also reduces the entropy of neural activity. Perception and action might be similarly coordinated by coherent phase relations among multiple neuronal spike rates.

Psychophysical evidence demonstrates that the visual system is indeed very sensitive to the coherence of spatiotemporal patterns (Lappin & Bell, 1976; Lappin & Craft, 2000; Lappin, Donnelly, & Kojima, 2001; Lappin, Norman, & Mowafy, 1991; Lappin, Tadin, & Whittier, 2002). Stereoscopic acuity in particular indicates that binocular vision must function as a coherent aperture (Lappin & Craft, 1997, 2000).

## Visual Information is Spatiotemporal

Two emphases throughout Gibson's research program on visual perception were that (a) vision is an active process by moving observers, and (b) the image transformations produced by moving observers and moving objects are basic forms of information about spatial structure. Gibson's observations in 1950 about the informational significance of the geometry of changing spatial patterns in the eyes of moving observers were major insights. Previously, the idea that such geometrical transformations could be information about spatial structure was barely imaginable.

For most experimental psychologists of that era, space and time were separate physical dimensions. Retinal images had two spatial dimensions. And a third dimension in depth was necessarily an inference from “cues” such as occlusion, linear perspective, and motion parallax.^3^ Motion perception and form perception seemed functionally separate.

Information in general and information for visual perception in particular is based on invariance under transformations. A vast collection of experimental evidence over the past 50 years clearly illuminates the fundamental nature of spatiotemporal structure at all levels of visual perception. Rogers’ (2021) paper “Optic flow: Perceiving and acting in a 3-D world” in this special issue describes Gibson's influence on our current understanding of perception of three-dimensional (3-D) structure from motion. Landmarks in that development include Johansson's studies of relative 2-D motion perception (1950/1994) and of biological motion perception (1973), Braunstein’s (1976) monograph on *Depth Perception through Motion*, and Rogers and Graham’s (1979) study of motion parallax. No one who has seen those vivid demonstrations can still believe that space and time are visually independent. Indeed, vision is more sensitive to spatial relations in moving than in stationary patterns (Lappin & Craft, 2000; Lappin, Bell, Harm, & Kottas, 1975; Lappin, Donnelly, & Kojima, 2001; Lappin, Tadin, & Whittier, 2002). And our nervous systems evidently lose almost no information about image motion from the retina through the cortex (Borghuis, Tadin, Lankheet, Lappin, & van de Grind, 2019).

## Surface Structure is a Basic Form of Visual Information

The dynamic structures of environments, optical images, neural networks, and conscious experience have distinctly different spatial dimensions, spatial and temporal frequencies, energies, material elements, and structural features. What variations in these diverse domains can explain their corresponding spatiotemporal structures?

One aspect of that problem now seems understandable, at least in broad outline: Surface structure is a basic form of visual information. The spatiotemporal structure of 2-D images corresponds to the 3-D spatial structure of environmental surfaces.

Gibson emphasized the critical role of surfaces in his (1979) ecological approach. His nine “ecological laws of surfaces” include the following (pp. 23–24):

- *All persisting substances have surfaces, and all surfaces have a layout.*
- *Any surface has a characteristic shape, . .*.
- *Any surface has a characteristic texture, depending on the* composition *of the substance*.
- *An illuminated surface may absorb either much or little of the illumination falling on it*.
- *A surface has a characteristic reflectance, depending on the substance*.

These were insightful points, well ahead of visual science at that time. Subsequent research has supported and elaborated these ideas. We now know that information for perceiving the spatial shape, colors, and substances of the environment involves the optical image structure of surfaces. Perceived environmental surfaces are not inferred from simpler visual “cues”, but correspond directly to the spatiotemporal optical images of surfaces.

Photos of common scenes and objects in Figure 2 may suggest some of the detail about the 3-D shapes and substances of environmental objects provided by even stationary images. Moving one's vantage point within such 3-D scenes adds basic information by the structural invariance of transformations over multiple images, as discussed below.

**Figure 1.**
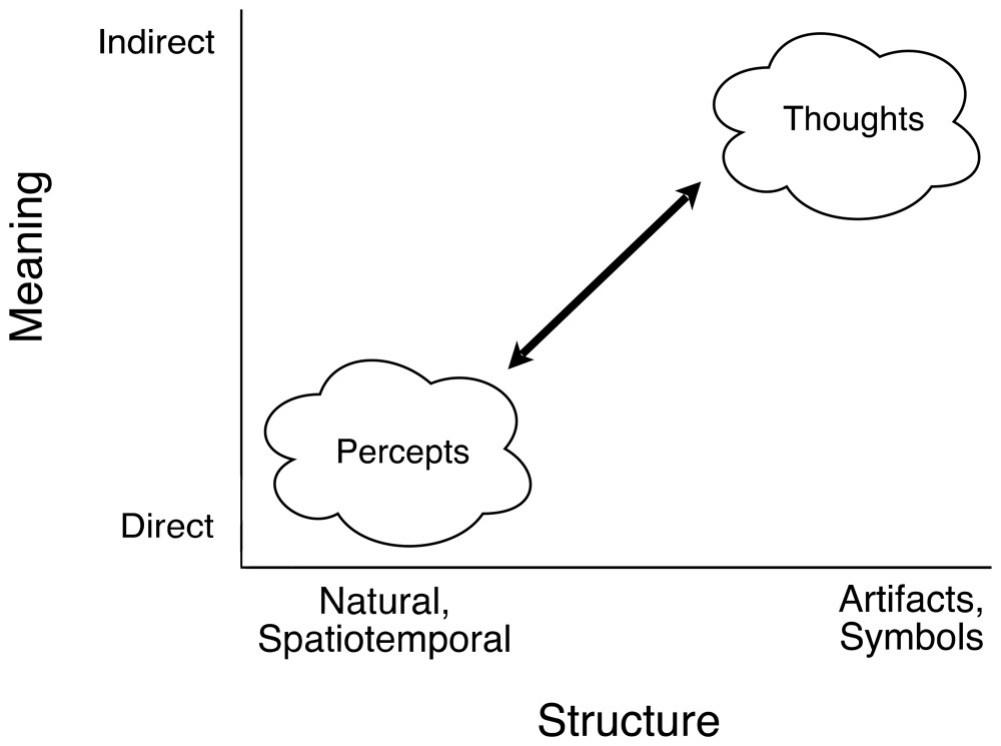
A psychophysical continuum of varied forms of information, from natural, spatiotemporal structure to human-made artifacts and symbols. All information is based on corresponding relational structures of variation in physically separate domains. And the relevant relational structure of any given domain is defined by the transformations under which that relational structure remains invariant and preserves correspondence with another domain. The natural spatiotemporal structures pertinent to Gibson’s ecological approach are invariant under environmental changes in context and illumination and observational changes in the observer's vantage point and motion. Symbolic information relies on consistencies of usage in social groups. Thus, spatiotemporal structure of natural information is directly meaningful, but the meaning of symbolic information derives from the observer's socially based knowledge. This illustration is based on a diagram of Gibson (1966, p. 244) on “the difference between perceptual meaning and verbal meaning”.

**Figure 2.**
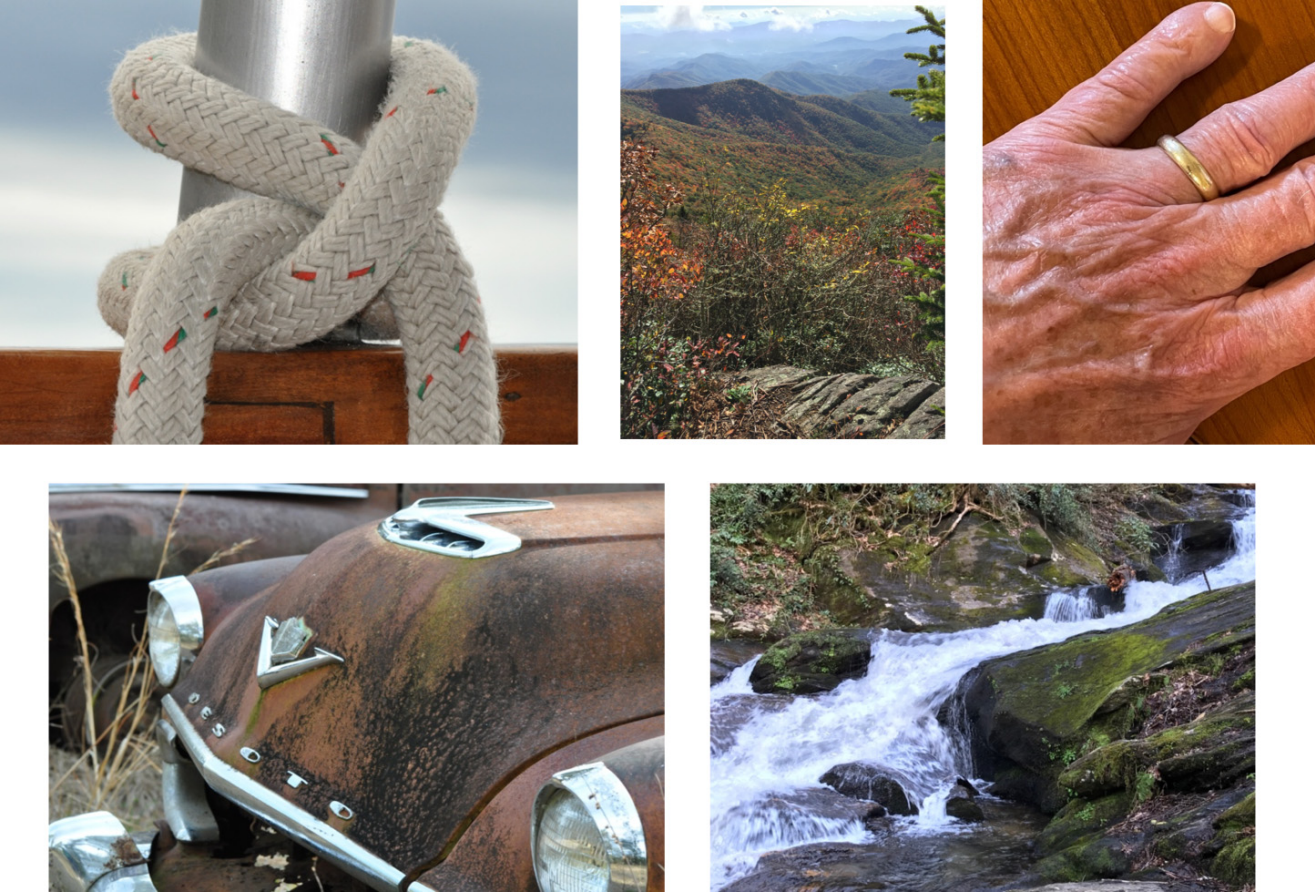
Five photographs of everyday objects and scenes that may illustrate the rich visible detail in even stationary image information about the local shapes and material substances of environmental surfaces. The shape at each local patch on a smooth (differentiable) surface is either (a) elliptical, convex, or concave (hills and valleys), where the minimum and maximum curvatures have the same sign; (b) hyperbolic (saddle-shaped), where the minimum and maximum curvatures have opposite signs; (c) parabolic (cylindrical), where one direction is curved and the other is not; or (d) planar, where the surface is not curved in either of the two directions. Elliptical and hyperbolic regions are separated by parabolic lines, where a principal curvature changes sign. The curvature in any given direction on a smooth surface is defined by the rate of change in direction of the surface normal relative to change in surface position — a second-order spatial derivative. Directions of minimum and maximum curvature are orthogonal. The ratio of the two principal curvatures is an intrinsic property of the surface, independent of a three-dimensional (3-D) reference frame, and defined in two-dimensional (2-D) images. The magnitude of curvature in a given direction, however, is not defined in the image. These photographs also illustrate interactive relations between perceived colors and material substances. Surface microstructure affects macroscopic image structure by the way it affects the scattering of incident light. Material substance, spatial structure, and wavelength spectrum interact. Gold and silver colors, for example, do not appear in the rainbow. Nor do the color qualities of wood, skin, rocks, metal, water, etc. (The photos in this illustration were by the first author, JSL.)

**Figure 3.**
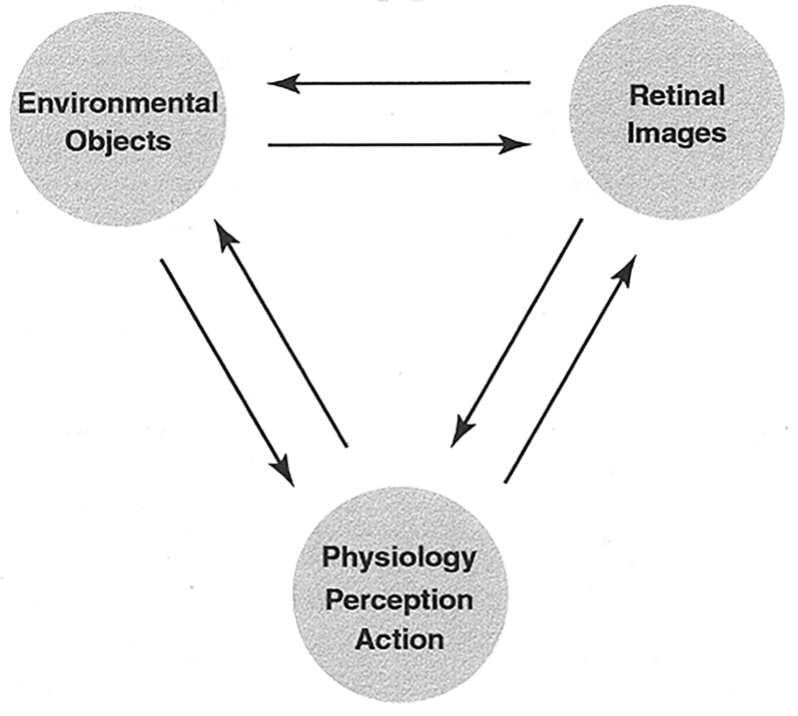
A schematic illustration of the approximate isomorphism of the spatial differential structures of environmental surfaces, retinal images, and perceptual systems in the visual system, brain, and experience. These correspondences have been tested and supported in psychophysical experiments. This conceptual illustration is oversimplified in that correspondences may be noisy and selective. (From Lappin & Craft, 2000, p. 8.).

**Figure 4.**
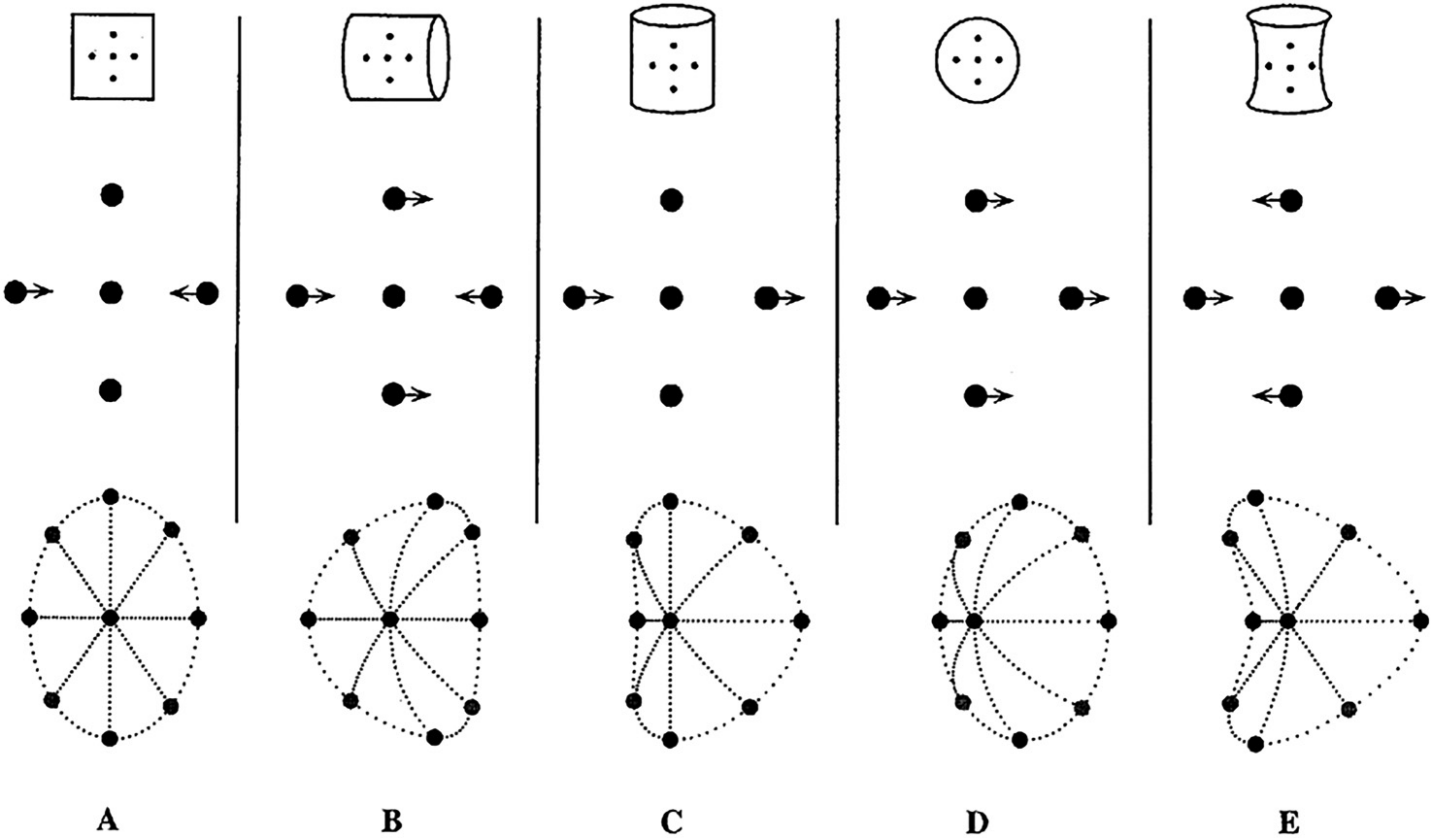
A schematic illustration of the qualitative second-order differential structure of the two-dimensional (2-D) neighborhood of a given point in the inter-image displacement fields associated with stereoscopic disparity or rotation in depth for each of the four possible local shapes of a smooth surface. **A**: planar; **B** and **C**: parabolic (cylindrical) in two different orientations; **D**: elliptic (concave or convex, bumps and dimples, hills and valleys); **E**: hyperbolic (saddle-shaped). The central point in each pattern is a stationary reference point for describing the surrounding image displacements. (These diagrams were suggested by Steven Tschantz, Department of Mathematics, Vanderbilt University.) (From Lappin & Craft, 2000.).

## Information about surface shape is intrinsically 3-D

Contemporary understanding of the geometrical structure of surfaces and their images owes much to the contributions of Koenderink and van Doorn, who clarified correspondences between the differential geometry of environmental surfaces and that of their spatiotemporal images (e.g., Koenderink, 1984a,b, 1987, 1990; Koenderink & van Doorn, 1975, 1976a, b, c, 1992a, b). Two-dimensional images of surface shape constitute direct information about the intrinsic three-dimensionality of the surface. Geometric properties that define the local shape at each visible point on an environmental surface are also available in the optical images, and these properties are invariant under the geometric transformations produced by relative movements of the object and observer in 3-D space.

Surface shape is defined at every point by the second-order differential structure of 3-D spatial relations over the surface. The second-order differential structure of the image and the second-order structure of the environmental surface correspond to one another—especially when there are multiple images of the object, due to binocular vision or relative rotation of the observer and object. For such spatiotemporal images, surface shape information is given by a *diffeomorphism* between the environmental surface and its retinal image structure (see references above for Koenderink and Koenderink & van Doorn; also Lappin & Craft, 2000; Lappin, Norman, & Phillips, 2011). Additionally, the self-occluding boundary contours of smooth surfaces are highly visible specifications of the surface shapes at those locations (Koenderink 1984a, b).

Psychophysical experiments have also shown that (a) human vision is highly sensitive to the geometrical properties that define surface shape, and (b) this visual sensitivity is robust over transformations of lower-order image properties—translation, expansion, slant, tilt—associated with relative movements of the object and observer (e.g., Lappin, 2014; Lappin & Craft, 2000; Lappin, Norman, & Phillips, 2011; Perotti, Todd, Lappin, & Phillips, 1998; Phillips & Todd, 1996; Phillips, Todd, Koenderink, & Kappers, 2003; Todd, 2004). Thus, the visual system and brain receive direct information about the 3-D shapes of surfaces from retinal images. Visual perception of 3-D surface shape is based directly on the second-order differential geometry of retinal patterns. It is not an inference from lower order elements.

Nevertheless, the images do not determine the scale of objects’ extensions in depth. And the depth scale is not reliably perceived (e.g., Norman & Todd, 1993; Todd, 2004; Todd, Oomes, Koenderink, & Kappers, 2004). As Todd (2004) pointed out, however, the set of potential shapes corresponding to a given image is highly constrained. A one-to-many mapping from image to objects is consistent with precise visual information because the set of possible objects that correspond to a given image is quite restricted.

### Shape is spatial information rather than stimulation

The distinction between shape as *information* versus stimulation—as relational structure versus energy—can be demonstrated by experiments that evaluate shape discrimination by vision, haptics (active touch), and cross-modal comparisons. Gibson (1963) referred informally to such demonstrations without the specific data, but Farley Norman and Flip Phillips (Norman et al., 2004; Norman et al., 2012; Phillips et al., 2009) have carefully evaluated haptic shape discriminations, using both natural objects (bell peppers) and replicas of Gibson's “feelies” (10 small solid sculptures). The general results are clear: solid shapes can be discriminated with similar accuracy visually, haptically, and by cross-modal comparison. Thus, shape is a basic form of information—invariant with changes from optical to mechanical energy.

### Shape, texture, reflectance, color, and material substance are covariant and interactive aspects of image information about environmental surfaces

Optical images are reflections from surfaces. The energy and spectral composition of images are jointly determined by extrinsic illumination and intrinsic reflectance of the surfaces. Long-standing questions concern the optical information that yields perceptual constancies of object lightness and color despite changes in ambient illumination.

Related questions concern the perceived material substance of objects. The trichromatic theory of color vision is well established: To an excellent approximation, the perceived color of light of any wavelength composition can be visually matched by a linear mixture of red, green, and blue primary components. How, then, can this theory be insufficient to describe the perceived colors and substance of objects such as metals (gold, silver), glass, wood, skin, fur, rocks, fabrics, plastics, vegetation, paper, etc.—as seen in Figure 2? What is the optical image information for perceiving material substance?

These questions arise within the science of ecological optics that Gibson presciently outlined in his 1966 and 1979 books. In the present special issue on Gibson's *ecological approach*, Todd (2020) gives an excellent history, introduction, and survey of contemporary psychophysics of ecological optics. The reader is directed to that paper as background framework and illustration to supplement the present brief discussion.

The reflectance of light at each point on the surface of a solid object is specified by its bidirectional reflectance distribution function (BRDF) — the percentages of radiant energy of given wavelengths from given directions reflected in given directions (Nicodemus et al., 1997). In one sense, the physics and geometry are simple, but the possible combinations of specific parameters are unlimited. The range of potential illumination directions at a given point on an opaque surface is a hemisphere above the tangent plane at each surface point; and the percentages of light of given wavelengths reflected in varied directions from that point are distributed over a similar hemisphere of directions. Thus, the BRDF of a given object depends on its material composition, which affects its wavelength-dependent reflectance, absorption, and spatial scattering of light in varied directions above the surface and also below in the case of translucent materials. The BRDF is the optical information about the color and material substance at a given surface point on a given object. It is independent of the object's shape and illumination.

Optical images, however, are images of potentially moving objects in the eyes of often-moving observers. The image of a stationary object in the eye of a stationary observer or camera is a temporally frozen spatial pattern of luminous energy and wavelength composition — for simplicity, patterns of ‘shading’ and ‘color’. These shaded colored images are the combined effects of three sets of factors—*photometric variations* in the intensities, wavelengths, and directional distribution of illumination at each point on the surface, *geometric variations* in surface shape and orientation relative to the directions of both illumination and image position, and the *BRDF pattern* determined by the object's material substance. What, then, is the image information that enables perception of the object's shape, color, and material substance? How do we distinguish an object's shape from its color and material substance? What optical image properties are robust under variations in illumination and viewing direction?

At least partial answers are offered by a basic insight recently clarified and evaluated in elegant psychophysical experiments by Marlow and Anderson (2021): The photometric, geometric, and material factors have correlated and interactive effects on image structure. Surface shape and reflectance characteristics covary and are jointly specified by certain “photogeometric” constraints on images.

Marlow and Anderson investigated two such constraints: (1) The surface shape and reflectance of an object are constrained and specified near self-occluding boundary contours. Because image shading due to surface reflectance vanishes at the boundary contours, external effects of illumination, shadows, and occluding objects are segregated from effects of surface reflectance at the boundary contours (Anderson & Winauer, 2005; Marlow, Mooney, & Anderson, 2019). And the 2-D curvature at each point on the boundary contours identifies the 3-D curvature and shape: positively curved contours identify convexities, negatively curved contours identify saddle-shaped regions, and straight contours identify edges of cylindrical or planar regions (Koenderink, 1984a, b). 2. Correlated variations in surface shape and image shading gradients also distinguish convexities versus concavities and translucent versus opaque materials. Specular highlights occur at points of highly curved convexities of reflective surfaces and run along lines of minimal curvature, outlining bumps and dimples (Fleming, Torralba, & Adelson, 2004; Todd, Norman, & Mingolla, 2004). Marlow and Anderson (2021) showed in addition that image shading gradients due to the surface orientation and convexity versus concavity are systematically different for opaque and translucent materials—translucent materials producing shallow shading gradients and more homogeneous shading at concavities.

A third form of optical information about material substance and color involves the scattering of light. Large effects of surface microstructure on macroscopic image shading were described by Tadin, Haglund, Lappin, and Peters (2001), who quantified the light scattering distributions of roughened silica glass and correlated these with the root mean square (RMS) roughness of the microscopic surface topography on scales comparable to the wavelength of light—from about λ/10 to 10λ. Unroughened or slightly roughened glass was transparent, but greater roughness produced highly frosted opaque surfaces without visible specularity. The shift from specular to broad scattering was abrupt, occurring at RMS roughness approximately equal to the wavelength of light. Thus, relative roughness was greater for green than for red light and scattering of green light was both greater and qualitatively different from that of red light. This microscopic texture is invisible, but it has fully visible effects on image shading gradients and probably perceived color as well.

Evidently, optical information about the rich variations of environmental surfaces—with characteristic shapes, colors, lightness, opacity, textures, and material substance—involves correlated and interactive effects on images. And this information about environmental objects seems to be robust under variations in illumination and viewing direction. The information is optical, not inferential.

## Brief Comments About Information for Perceiving and Navigating 3-Dimensional Space

Visual space is often conceived as an abstract, extrinsic reference frame that is independent of the objects it contains. The intuitive 3-D space of our experience and actions is often regarded as an abstract empty framework inferred from 2-D retinal images, depth cues, and statistics of past experience. Gibson rejected such concepts of abstract visual space as a fundamental error (e.g., 1979, pp. 3, 149).

Gibson suggested that spatial layout of the ground plane and other environmental surfaces must constitute intrinsic spatial information about depth. As emphasized in a previous section, however, the depth scales of solid objects and of separations between objects are not determined by their retinal images, even in moving and stereoscopic images. The empirical unreliability of depth discriminations contrasts with the subjective certainty and clarity of our visual experiences and intuitions about environmental spaces.

A salient characteristic of the long history of research on space perception is its inconsistency, both empirically and theoretically. Perceived spatial relations vary with task demands, attention, and scene context, and are often inconsistent among different vantage points and even among different aspects of the same object (Lappin, 2016; Lappin, Shelton, & Reiser, 2006). Visual awareness is ordinarily very tolerant of such inconsistencies, but they can be quite visible if one attends to them. Koenderink (2001) pointed out that such inconsistencies are commonplace and concluded that they are evidence for a “multiple-visual-worlds hypothesis”.

### Active vision and navigation

As reviewed and discussed by Rogers (2021) and Warren (e.g., 2006, 2021), Gibson emphasized from 1950 onward the importance of interactive coordination of vision and locomotion. Our skills in moving through often-crowded and changing environments demonstrate the power of visual information for guiding our movements. And the optic flow that results from our actions gives continuing visual feedback about egocentric directions and relative distances. Spatial information from optic flow is not metric, but evidently it need not be for most visually guided locomotion.

Gibson's early analyses laid the foundation for a new psychophysics of space perception. One of his early insights was that optical information for guiding locomotion is egocentric, defined in relation to the individual observer. The observer-specific information differs from that of other observers, and also from what might be seen in a God's-eye view by an external scientist. For a pilot landing a plane on a runway, for guiding one's steps along a winding rough forest path, or for a baseball player running to catch a fly ball, the optic flow is not only different from that of an external observer, it is also simpler—specified by optic flow in the 2-D space of the actor's eye rather than in the 3-D space for an external observer. Warren (2021) also points out that the optic flow for navigating around obstacles and through openings is structured and scaled relative to the observer's actions as well as her eyes.

Movements in three dimensions are combinations of translations (forward/backward, left/right, up/down) and rotations (roll, pitch, yaw) along three perpendicular axes. These movements by either the observer or objects transform the optical array in a “lawful [manner] which leaves certain properties . . . invariant” (Gibson, 1950, p. 153). One of Gibson's insights was that the invariant visual information may be simplified as a dyadic spatial relation between a specific spatial target and the viewpoint of the moving actor whose motions are intended to intercept or collide with the target. Visual information for controlling locomotion in such cases is based on optic flow at the actor's viewpoint, with continuing feedback between the movements and the flow field.

Consider the visual control problem for landing an aircraft. A critical part of this task is to maintain the correct glide path so that the aircraft will touch down at the desired target point on the runway. If the glide path is too shallow, the aircraft will land too far down the runway, and if the glide path is too steep, the aircraft will touch down hard in front of the desired aim point with risks in hitting objects or terrain. Gibson's analysis revealed the relation between glide path and the structure of the expanding optic flow around the pilot's aim point. If the glide path is too shallow, the expansion point moves upward toward the horizon, but if the glide path is too steep, the expansion point moves toward the pilot. Gibson pointed out that the same pattern of expansion is available to both the novice and the experienced pilot. The experienced pilot does not see more than the novice, but has learned to discriminate relevant differences in the optic flow.

This particular visual control problem is but one example in a large class of problems pertinent to biology, engineering, and sports as well as the visual sciences. A large and growing research literature has identified numerous lawful relationships that constitute information available to active perceivers as they encounter static and moving objects. Examples include controlling time-to-collision (e.g., Boostsma & Oudejans, 1993; Gray & Regan, 1998; Lee, 1976), intercepting a moving target (e.g., Zhao & Warren, 2017), and controlling the altitude of moving aircraft (e.g., Kleiss, & Hubbard, 1993; Warren, 1988).

## Limited Rates and Capacities of Human Perception

The preceding discussions dealt with the nature and identification of visual information, which was the focus of Gibson's ecological research. Another basic aspect of information, however, involves quantification. If individuals’ perceptions of their environments are quantitatively limited, then such limitations are clearly fundamental for visual ecology. How might perceptual information be quantified?

Shannon's (1948; Shannon & Weaver, 1949) mathematical theory of communication contributed an elegant method for quantifying both (a) the amount of information given by choices from sets of possible messages and (b) the rate at which such information is transmitted over a given communication channel. From Gibson's perspective, Shannon's symbolic definition of information was a critically misleading way to describe the spatiotemporal information for perceiving and acting. From the perspective of the information-processing paradigm, however, not recognizing perceptual and cognitive limitations was a basic shortcoming of the ecological approach. Those mutual critiques both had some validity.

A principal challenge in measuring perceptual capacity has involved quantifying environmental information. However, environmental information is not objectively defined, and that was one reason for Gibson's dissatisfaction with the concept of information (e.g., 1979, pp. 62–63). After the following explanation, we will suggest an alternative approach.

### Visible information is not objectively quantifiable

Information is based on corresponding relational structures. The psychophysical structures for Gibson's ecological approach are primarily spatiotemporal. For multiple reasons, such potential information is not objectively defined.

In the first place, visible information is egocentric—defined relative to an individual observer. Optic flow in the eye of an individual actor is both different and simpler than that seen by an outside observer.

Second, active observers select (“pick up”) information. Selected information depends on the observer's goals and actions at a particular time and place, on knowledge of the context and meaning, and on the relevance of spatiotemporal forms and symbols in that setting to the observer's current interests, attitudes, and state of mind and body.

Third, the set of all possible spatiotemporal and symbolic relationships that might constitute visible information sometime someplace for someone for some purpose is practically unlimited. Potential information is certainly not arbitrary—it involves invariants over time, place, context, and so forth—but it depends on its relevance to particular discriminations and actions.

Fourth, information often also involves prior uncertainties. However, potential observations in unrestricted natural settings are often unknowable beforehand.

An alternative: Even if the input information is not objectively quantifiable, the rate of perception might be quantified by the rate of an observer's output behavior in coordinating actions with environmental variations.

### Shannon's fundamental theorem about channel capacity

The fundamental theorem of Shannon's (1948; Shannon & Weaver, 1949) theory of communication states that for any given channel of communication there exists an upper limit on the rate at which it can transmit information. This is a physical limit, imposed by that channel's maximum rate of entropy. Shannon defines the rate of information transmission, in bits/s, as

R=[U(X)–U(X|Y)]/t=[U(Y)–U(Y|X)]/t,

where *U(X)* and *U(Y)* are the input and output uncertainties, respectively, and

$$U(X)=\text{–}\sum_{i}[\;p(x_{i})\;\mathrm{lo}g_{2}p(x_{i})]$$

is the average uncertainty of the input information, *U(Y|X)* is the conditional uncertainty of the output given the input, and *t* is a time interval in seconds. Accordingly, the channel capacity, *C*, is the maximum value of that rate,

C=Max[U(Y)–U(Y|X)]/t.

After the publication of Shannon's theory, experimental psychology used that theory to quantify human perception and performance. Opinions about its success vary, but that research discovered what seemed at the time to be principled methods for quantifying human perception and its limits (e.g., Eriksen and Hake, 1955; Fitts, 1954; Garner, 1962; Hyman, 1953; Miller, 1956). Experimental tasks typically evaluated accuracies of “absolute judgments” in identifying stimuli randomly selected from specified sets of alternatives. Capacities measured that way averaged over many studies at around 2.5 bits—the basis for Miller’s (1956) words about “the magical number seven, plus or minus two” (−log_2_ (1/7)  =  2.81 bits). Hyman (1953) found a linear dependence of response times on uncertainty with a slope of about 5.75 bits/s. That line of research, however, now has little influence, and Gibson regarded it as irrelevant to his ecological approach.

Shannon's quantification is quite general, applicable to physical signals of any form. However, it is a weak measurement that treats signals and messages as only nominal categories, involving merely *same*/*different* relations. Spatial and temporal order, similarity, difference, distance, connectedness, gradients, or so forth are irrelevant. The amount of information in such categorical structures is based entirely on statistical uncertainty, on the probability distribution over the set of possible signals and messages. In Shannon's communication model, the set of possible signals is known beforehand to both sender and receiver, but that prior knowledge is not realistic for perceiving and acting in most natural environments. Moreover, categorical structures are not much help in describing environmental surfaces and images, optic flow for actions in 3-D environments, or most other information relevant to ecological psychophysics.

### Measuring rates of discrimination and action

Despite the insufficiency of Shannon's methodology for identifying or quantifying environmental information, we recently found it to be useful for describing the output information rates for behavioral actions. Our experiments involved simple measures of response times (RTs) for recognizing specified targets. The rationale and computational methods are detailed in other papers (Lappin, Lowe, Reppert, Schall, & Bell, in preparation; Lappin, Morse, & Seiffert, 2016; Lappin, Seiffert, & Bell, 2020), so they are only sketched here.

Simple computations yield ratio-scaled measures (in bits/s) of visual recognition rates over time following a given visual event. Using factorial experiments, we quantified the effects of visual information format on the time course of recognition. The methods offer a strategy for measuring perceptual rates. And our findings point to quantifiable limits on the rates of perception.

A few basic formulas are useful before illustrating the methods.

- RT survivor function: S(t)=1–F(t)=P(RT>t).
- cumulative hazard function:^4^ $H(t)=\text{–}\mathrm{lo}g_{2}[S(t)].$
- information gained in a particular time interval [t,t+Δt):G([t,t+Δt))=H(t+Δt)–H(t).
- estimated hazard rate, recognition rate in a given interval: g(t)=G([t,t+Δt))/Δt.

These computations are simple numerical transforms of the rank-ordered RTs, but they measure the temporal processes of visual recognition. The cumulative hazard rate is a ratio scale of the information acquired by a given time, and the hazard rate measures the recognition rate at a given time. The hazard functions are non-parametric descriptive measures with no assumptions about the distribution parameters or stochastic processes. In several studies, the hazard rates revealed lawful characteristics of recognition processes that were not evident in the RTs as such. Lappin et al. (in preparation) summarize the converging results of several studies that validate the interpretation of these measures.

Evidence that visual recognition occurs at a limited rate was obtained in two studies by Lappin et al. (2016) and Lappin et al. (2020). In Lappin et al. (2016), the observers’ task was to rapidly detect a nonrandom target motion by one of a set of otherwise identical objects moving in randomly changing directions. These target motions occurred unpredictably during continuous observation periods of several minutes. To detect the target, one had to discriminate the nonrandom target motion from the random-motion background. Target motions continued until they were detected, becoming more visible with increasing duration and distance. Variations in target uncertainty and visual field complexity were based on the set size, *n* *=* {2, 4, 6, 8}, of moving objects.

**Figure 5.**
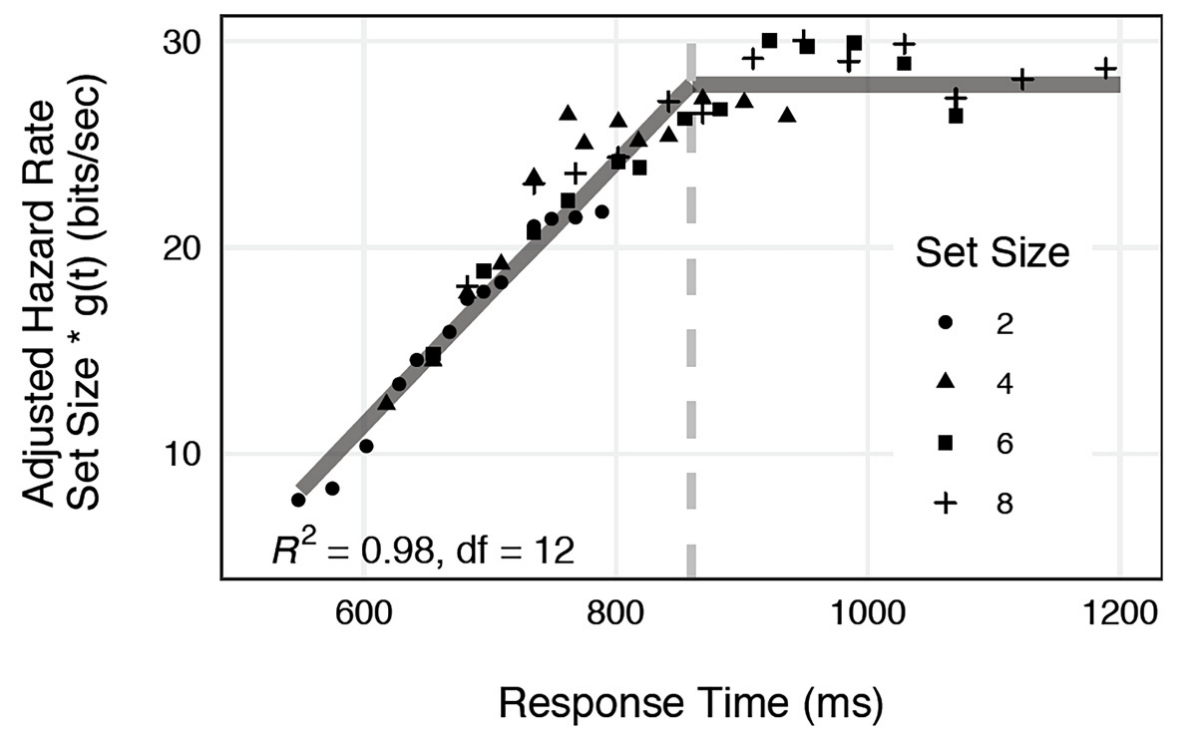
Adjusted hazard rates, *n* × *g_n_(t)*  *=*  *n [H_n_(t* *+* *Δt)* *−* *H_n_(t)]/Δt*, as a function of response time (RT). Multiplying each hazard rate by the set size *n* estimates the rate for set size *n*  *=*  *1*, illustrating a common temporal process independent of set size. Successive points on each function were estimated in running 20% windows at successive 5% intervals of the survivor function, at *S(t)*  *=*  {0.90 *−* 0.70, 0.85 *−* 0.65, 0.80 *−* 0.60, . . . 0.30 *−* 0.10}. A total of 16 independent data points (four independent data points for each of the four set sizes, at successive 20% intervals) were described by three parameters, *R^2^ (df* *=* *12)*  =  0.98. The function given by those parameters is shown by the solid lines. (This figure is adapted from Lappin et al. (2016), Fig. 3, p. 2475.)

Figure 5 is a graph of the resulting hazard rates. To illustrate how the temporal structure of the visual process was invariant with set size, each of the rates has been multiplied by set size, thereby estimating performance for set size *n*  =  1.

As may be seen, the four hazard rate functions, *g_n_(t)*, at any given time were (a) inversely proportional to the set size, and (b) increased proportionally with RT over roughly 400–900 ms, independent of set size. Thus, hazard rates at any given RT were described as a numerical product of two functionally distinct factors—a visual process that gained information proportionally with time, invariant with set size, and a visual uncertainty factor proportional to set size, invariant with RT:

$$g_{n}(t)=V(t)\;∙\;U{\left(n\right)}^{\text{–}1}∙\;C,$$

where *V(t)* represents visual integration of motion information, proportional to time over an interval of roughly 0.5 s [*V(t)*  *=*  *(t − c)/(k − c)* for *c* *≤* *t* *≤* *k, V(t)*  *=*  *0* for *t* *<* *c,* and *V(t)*  *=*  *1* if *t* *>* *k,* with *c ≈ 0.4 s* and *k ≈ 0.9 s*]; *U(n)*  *=*  *n* represents the target uncertainty and visual field complexity; and the constant *C* ≈ 28 bits/s represents a limiting *channel capacity*—the estimated maximum recognition rate for set size *n*  =  1.

Thus, the rate of visual recognition at any given time was determined by three functionally separate factors: (a) temporal integration of visual motion information, (b) a divisive effect of visual field complexity, and (c) a limiting recognition rate resembling Shannon's maximum channel capacity. With those three parameters, the above equation accounted for 98% of the variance in 16 independent hazard rates (4 for each set size).

Converging evidence of a limiting channel capacity was obtained in a second study by Lappin et al. (2020), who also used hazard rates to evaluate effects of visual field complexity. Temporal characteristics of the visual information, the detection task, and RT distributions were all different from those of the previous study. The moving patterns were more predictable, involving constant-speed linear trajectories representing “flight paths” of a number (*n*  =  {1, 2, 4, 6}) of “planes” of various colors that identified “friend”, “foe”, or “unknown” status. The observer's task was to detect a sudden change in either the color or motion direction of a randomly selected plane. In a simple detection task, the observer was required to respond quickly to a change in either color or motion direction; and a selective detection task required a response only when the change indicated an increased “threat” defined by half of the color changes and half of the direction changes.

The principal result pertinent to the present discussion was that increased set size again had a temporally constant divisive effect on the hazard rates. That effect was also constant over performance differences due to changes of color versus motion direction and over differences between simple versus selective detections. These time-invariant effects of visual field complexity were striking because the recognition rates for visual changes varied markedly over time, resembling inverted V-shaped functions of a brief window of awareness that opened and closed over 200–300 ms after a display change, after which the sudden visual changes were no longer noticed. Changes in motion direction were less detectable than color changes, selective detections were less frequent than simple detections, and both of these effects varied with time. Nevertheless, the temporally constant divisive effects of visual set size were independent of both of those variables. Recognition rates (RT hazard rates) at any given time were again selectively influenced as products of functionally separate factors associated with (a) visual field set size, (b) type of visual change, (c) detection task, and (d) a maximum rate parameter, *C* ≈ 21 bits/s.

Together, these recent findings suggest that Shannon's fundamental theorem — that any physical channel has a maximum rate at which it can transmit information — probably applies to the rate of conscious visual recognition. The visual nervous system is, after all, a dynamic, energy-consuming physical system. Perhaps we should expect that visual perception will also occur at a limited rate. Evidently, spreading visual attention over multiple objects and events reduces the rate of perceiving any individual thing.

James Gibson saw Shannon's mathematical theory as irrelevant for characterizing the spatiotemporal forms of information relevant to the ecological approach. His doubts were well-founded. Accordingly, questions about possible limitations on the capacity and rate of perception have been largely overlooked in the ecological approach. Nevertheless, perceptual capacity is relevant to the dynamic relationships between environments, observers, and actors.

Perceptual capacity has long been important in experimental psychology (e.g., James, 1890; Woodworth & Schlossberg, 1954; Wundt, 1894) and in the information-processing approach (e.g., Miller, 1956; Norman, 1969; Schneider & Shiffrin, 1977; Sperling, 1960). Visual information in those research paradigms, however, has typically involved varied numbers of briefly viewed, discrete, static ‘stimuli’ rather than the spatiotemporal patterns relevant to the ecological approach.

Thus, a major open area for research concerns the rate at which vision acquires spatiotemporal information in real time. The ecological approach has often investigated task environments that demand real-time spatial and temporal coordination of an action and an environmental event (e.g., Warren, 2006; Zhao & Warren, 2017), though the actions have usually focused on single well-defined events. Insufficient evidence is available about how the complexity of environmental information affects the rate of perception. We still know too little about the perceptual complexity of information in natural spatiotemporal patterns involving optic flow, 3-D spatial relations, surface shape, and photo-geometric effects. Better understanding of perceptual capacity limits is important not only for the ecological approach, but for psychological and brain sciences more generally. Questions about perceptual capacity also have widespread applications in human factors engineering, education, sports, and medicine.

## Notes

1. Input and output information for many computer engineering applications is spatiotemporal, however—for example, in robotics, control systems for moving vehicles, signal processing, and image interpretation.

2. Current understanding has changed. First-order spatial variations in texture do not specify the magnitude of surface slant. The spatial scale in depth is ambiguous from that image information.

3. The idea that image motion of near objects is faster than that of far objects is only half true. It depends on the point of fixation or reference.

4. Log_2_ is used merely for convenience of terminology, giving rates in bits/s. Literature on hazard functions uses natural logarithms, but the base of the logarithm affects only the scale units, not the meaning of the measures. The median RT is the time at which *H(t)* = 1.0.
